# Influence of Personality and Differences in Stress Processing Among Finnish Students on Interest to Use a Mobile Stress Management App: Survey Study

**DOI:** 10.2196/10039

**Published:** 2019-05-13

**Authors:** Mari Ervasti, Johanna Kallio, Ilmari Määttänen, Jani Mäntyjärvi, Markus Jokela

**Affiliations:** 1 VTT Technical Research Centre of Finland Ltd Oulu Finland; 2 University of Helsinki Helsinki Finland

**Keywords:** mental health, mobile applications, psychological stress, personality, neuroticism, mobile phone, surveys and questionnaires

## Abstract

**Background:**

Excessive stress has a negative impact on many aspects of life for both individuals and societies, from studying and working to health and well-being. Each individual has their unique level of stress-proneness, and positive or negative outcomes of stress may be affected by it. Technology-aided interventions have potential efficacy in the self-management of stress. However, current Web-based or mobile stress management solutions may not reach the individuals that would need them the most, that is, stress-sensitive people.

**Objective:**

The aim of this study was to examine how personality is associated with stress among Finnish university students and their interest to use apps that help in managing stress.

**Methods:**

We used 2 structured online questionnaires (combined, n=1001) that were advertised in the University of Helsinki’s mailing lists. The first questionnaire (n=635) was used to investigate intercorrelations between the Big Five personality variables (neuroticism, extraversion, openness, agreeableness, and conscientiousness) and other stress-related background variables. The second questionnaire (n=366) was used to study intercorrelations between the above-mentioned study variables and interest in using stress management apps.

**Results:**

The quantitative findings of the first questionnaire showed that higher levels of extraversion, agreeableness, and conscientiousness were associated with lower self-reported stress. Neuroticism, in turn, was found to be strongly associated with rumination, anxiety, and depression. The findings of the second questionnaire indicated that individuals characterized by the Big Five personality traits of neuroticism and agreeableness were particularly interested to use stress management apps (r=.27, *P<*.001 and r=.11, *P*=.032, respectively). Moreover, the binary logistic regression analysis revealed that when a person’s neuroticism is one SD above average (ie, it is higher than among 84% of people), the person has roughly 2 times higher odds of being interested in using a stress management app. Respectively, when a person’s agreeableness is one SD above average, the person has almost 1.4 times higher odds of being interested in using a stress management app.

**Conclusions:**

Our results indicated that personality traits may have an influence on the adoption interest of stress management apps. Individuals with high neuroticism are, according to our results, adaptive in the sense that they are interested in using stress management apps that may benefit them. On the contrary, low agreeableness may lead to lower interest to use the mobile stress management apps. The practical implication is that future mobile stress interventions should meaningfully be adjusted to improve user engagement and support health even among less-motivated users, for instance, to successfully engage individuals with low agreeableness.

## Introduction

### Background

The word *stress* has often somewhat differing definitions if it is used in a scientific compared with a colloquial context. From a physiological perspective, it generally refers to the loss of homeostasis of an organism [1]. Stress may have negative outcomes if it lasts too long or is too strong. On the contrary, stress may be beneficial if it is experienced in moderation relative to individual stress-sensitivity (eg, personality) and contextual features. Balancing the stress faced from the environment is a vital challenge, and thus, it is not surprising that stress is a major issue for well-being, health, and even proper functioning of the individual [2].

Stress is often referred to as if it exists in the environment per se, although it is not typically an objective feature of a specific environment. Instead, stress reactivity consists of an individual’s perception of an environment and their reaction to it [3,4]. Some individuals are more prone to stress than others, thus enabling measurement of individual stress proneness via personality and other features as well as coping styles. Personality and other descriptions of thought and behavior styles do not exist in a vacuum; they often become more self-evident after we understand the real-world consequences arising from them.

### Personality and Stress Proneness

Personality and temperament are defined as characteristic patterns of thoughts, feelings, and behaviors over time and across situations [5]. These traits are relatively stable across age groups, cultures, and time [6]. In addition, mood inductions have little effect on personality inventory scores [7]. Personality has been shown to affect exposure to a stressor, influencing the nature and severity of stressor experiences [5,8,9].

Possibly, the most commonly accepted model for describing personality is currently the 5-factor model called Big Five [10,11]: it consists of 5 global factors of neuroticism (N), extraversion (E), openness to experience (O), agreeableness (A), and conscientiousness (C). In general, high scores of neuroticism are linked with emotional instability and anxiousness; high scores of extraversion are linked with socializing and impulsiveness; high scores of openness are linked with creativeness and artistry; high scores of agreeableness are linked with trustworthiness and co-cooperativeness; and high scores of conscientiousness are linked with being hardworking and vigilance [11].

Furthermore, people differ greatly in their stress proneness and coping styles. Lazarus and Folkman [12] have argued that the major determinant of coping responses is the perception of the situation by the individual. Coping has been described as “personality in action under stress” [13], and people have been shown to develop habitual methods of dealing with stressors [12]. The process of coping is seen to consist of efforts made to manage a stressful situation so that it becomes less stressful [12], and *coping strategies* are seen as categories of behavior in response to stressful events [14]. All Big Five traits have been shown to be associated with specific coping strategies [5]. In recent years, it has increasingly been proposed that different coping strategies actually overlap with personality dimensions, and thus, they should not be treated separately but rather as trait complexes. Furthermore, personality may influence the ability to implement the chosen strategy and its degree of effectiveness [5,8,9].

In general, the personality trait of neuroticism and rumination is known to be associated with negative, stress-related conditions such as depression and anxiety [15,16]. Of the key features of depressive symptoms and stress states resembling depression, 1 is often related to repetitive negative thinking. This constant rethinking of adverse events or thoughts is called rumination [17]. All people experience traumatic and stressful experiences in their lives, but some people will fixate on bad memories, thinking about them continually, thus creating a constant internal stress for themselves. Thinking about the stressful experience might seem like a way to reduce stress and anxiety, but in fact, ruminating tends to increase rumination itself.

The importance and influence of personality traits over an individual’s stress proneness, coping processes, health, and health behavior have been confirmed in several previous studies [18-20]. For instance, an earlier study [21] regarding the associations between personality and key health behaviors indicated that highly conscientious individuals had more positive health behavior, such as exercising. People with high levels of extraversion, on the contrary, were associated with risky health behavior, such as cigarette smoking and alcohol drinking. The personality trait of conscientiousness is established to be associated with outcomes that are dependent on persistently working on something over the long run, eg, education [22]. Ferguson [23] provided a comprehensive review and a theoretical model to better understand interrelationships of personality, coping, health, and health behavior.

### Tailoring to Increase Intervention Effectiveness

Psychological interventions are typically resource-intensive and thus expensive. Technological approaches for delivering psychological interventions may increase their access and sustainability by being, among other things, less costly. They may extend the effective time beyond a therapy session or even replace some of the other treatment options [24]. Hence, the potential of technology tools in self-management of stress seems considerable owing to their cost-effectiveness and scalability [25].

Especially, mobile phones hold a great promise as an intervention delivery tool because they enable deeper integration into users’ everyday lives [26]. Thus, it seems feasible to implement health interventions using mobile technology; participants have demonstrated adequate compliance with the treatment protocol and have described momentary interventions as a credible and acceptable form of treatment [27]. Earlier researches have already noted that mental health interventions delivered through mobile apps can be effective in treating a range of mental health problems, such as depression, stress, and anxiety [28]. The use of mobile apps has been shown to enhance the sense of comfort and acceptance of the intervention [29,30].

However, mobile intervention solutions have not sufficiently taken into account the differences between individual users. Tailoring refers to the system autonomously performing the tailoring of personally relevant content without any direct user intervention through applying a preselected self-management strategy [27]. Technology-based systems can automatically tailor intervention content based on characteristics or information provided by individuals during preintervention or momentary assessments. Tailoring and smooth interaction with daily life appear to be more important for long-term adherence and beneficial outcomes than a multitude of options [31,32]. It has been shown that tailoring the content of health messages based on individual characteristics can improve message acceptance and willingness to change [33,34]. Furthermore, personally relevant content and tools may lead to better adherence and more lasting effects, especially in preventive programs [35,36].

### Research Questions

Personality influences people’s decision-making processes, and information about personality can be utilized in designing health apps [30]. We selected study variables known to be associated with stress reactivity, everyday emotions, and outcomes of long-term stress. Our online questionnaires included questions about personality, other processing styles of everyday information (rumination, self-reflection, and self-directedness), depressive symptoms, anxiety, coping styles, health status, and interest in using stress management apps. Relevant personal information about coping, personality, and related variables could be useful in tailoring individual interventions for better self-management of stress [37].

Personality and other traits involving stress reactivity and stress proneness may be useful in understanding the following empirical questions that are relevant for interventions, especially mobile interventions: (1) who are the individuals reporting most health and mental health problems, (2) who are most likely to start participating in an intervention, (3) who are most likely to continue participating in an intervention, (4) who are the ones benefitting the most from interventions, and (5) how could feedback and other features be tailored according to individual differences. The research variables and research questions of the study were designed to answer, when suitable, as many of these questions as possible.

With this exploratory study, we were interested in finding answers to the following questions:

1. How are an individual’s personality and stress-related background variables associated with (1) each other and (2) health and mental health variables?
2. How are the individual’s personality and stress-related background variables linked to the interest to use stress management apps?

## Methods

### Data Collection

We approached our research questions in an exploratory way, starting from the existing research and theories and then examining how people’s personality is related to stress and to their interest to use apps that help in stress management. Data were collected via 2 online questionnaires. The study was performed in accordance with ethical guidelines and it was reviewed by the Ethics Review Board in humanities and social and behavior sciences of the University of Helsinki. Table 1 presents a summary of the data.

The Big Five personality variables were assessed using the Neuroticism, Extraversion, Openness-Five-Factor Inventory (NEO-FFI) [38]. NEO-FFI is a shorter version of the Revised NEO Personality Inventory, and it contains 5 subsections that assess the factors of neuroticism, extraversion, openness to experience, agreeableness, and conscientiousness. There are 12 items per subsection, for a total of 60 items. Participants were asked to indicate their response to each item on a 5-point Likert scale ranging from strongly disagree (1) to strongly agree (5). Higher scores indicated higher levels of each personality dimension.

Self-reported stress was studied with a single item stress question and a description of stress (translation: “Stress means a situation in which a person feels tense, restless, nervous or anxious or is unable to sleep at night because his/her mind is troubled all the time. Do you feel this kind of stress these days?”) [39]. Depressive symptoms were assessed using the Center for Epidemiologic Studies Depression Scale questionnaire [40], and anxiety was assessed using the Generalized Anxiety Disorder questionnaire [41]. Rumination tendency was explored through the questionnaire devised by Elliot and Coker [42].

Moreover, the online questionnaire included basic queries about background information (eg, gender, age, education, work, social, and health status). The following questions were asked regarding the perceived usage activity of mobile apps and interest in using novel mobile apps: “Do you have a mobile phone?,” “Do you consider yourself as an active user of mobile application?,” and “Are you interested in using novel mobile applications on a daily basis?”

**Table 1:** Summary of data collection methods, numbers of participants, and goals of studies 1 and 2.

Data collection method	Participants	Goal of study
Study 1: structured online questionnaire	635 university students	To gain knowledge of the links between personality traits and stress-related mental disturbances such as depressive symptoms, anxiety, and rumination
Study 2: structured online questionnaire	366 university students	To explore how people’s interests for usage of technology-aided stress management solutions are related to their personality, stress proneness, depressive symptoms, and rumination

The second online questionnaire was otherwise similar, but it was somewhat improved by adding a few questions based on insights gained through the first survey round. The questions added to Study 2 were the following: “What kinds of stress management or relieving methods you are using?”, “Do you have previous experience on well-being applications?”, “If yes, what kind of application you have used?”, and “Are you interested in using a self-management application for managing stress?”

Apart from the previously mentioned items and questionnaire, Siegrist’s work stress measure (effort-reward imbalance, ERI) [43] was included in the questionnaires but was not used in this study. The sample consisted of mostly full-time students, the resulting ERI was less suitable for the sample. In addition to having few people actually reporting their work stress, the persons who reported the ERI were not likely full-time workers in professions that would match their educational background. Thus, the ERI was deemed as not suitable for this study.

The quantitative analysis of the questionnaire data was performed with IBM SPSS Statistics software. The Pearson correlation coefficient was used in the analyses.

### Study 1: First Online Questionnaire

The structured online questionnaire was used with the purpose of reaching a large sample of respondents representing a fairly heterogeneous group of individuals. Participants (n=635) were voluntarily recruited from the University of Helsinki, Finland, by sending an invitation via email to several of the distribution lists of university students in November 2014. The invitation contained a link to an electronic questionnaire, which was provided by the University’s Web service, *E-lomake*. Over 99% of the respondents responded within 14 days of the questionnaire being accessed.

Most of the respondents were female (86.9%; 552/635) and full-time students (79.5%; 505/635). Most of the respondents were 26 years or younger (69.0%; 438/635), and the age range was 18 to 62 years. All respondents responded in Finnish. It may be useful to clarify that there are practically no ethnic minorities among the Finnish-speaking students in the University of Helsinki; apart from Swedish-speaking Finns and potentially adopted children, there were no minorities in the sample or their number was extremely small (<1%). Thus, no further information regarding ethnicity is provided.

The socioeconomic background of the sample was relatively evenly spread; 25.4% (161/635) reported their family background as *working class*, 49.0% (311/635) as *middle class*, 23.6% (150/635) as *upper middle class*, and 1.9% (12/635) as *upper class*. Of the whole sample, 23.3% (148/635) had had a diagnosis of clinical depression at some point in their lives and 7.2% (46/635) were currently clinically depressed. Altogether, 14.3% (91/635) of the respondents reported having worse or much worse health than average people of their age, whereas 33.4% (212/635) reported having better or much better health than the average person of their age. Moreover, 22.5% (143/635) of respondents reported having difficulties *quite often* or *often* in making ends meet (=not having enough money). Of the respondents, 14.0% (89/635) reported being *rather dissatisfied* or *very dissatisfied* with their lives.

More than half of the respondents considered themselves as active mobile app users (56.1%; 356/635), and nearly half were interested in using novel mobile apps on a daily basis (49.1%; 312/635).

### Study 2: Second Online Questionnaire

The second online questionnaire (n=366) was similar to the Study 1 questionnaire except for the 4 supplementary questions concerning stress management and experience of well-being apps as presented in the Data Collection section. The data collection and analysis methods were similar to the first study. Email invitations were sent in February 2015. Over 97% of the respondents responded within 14 days of opening the questionnaire.

Most of the respondents were female (83.9%; 308/366) and full-time students (74.8%; 274/366). The age range was 19 to 56 years, with the majority of respondents being 30 years or younger (n=304; 83.0%; 304/366). Slightly over half of the respondents considered themselves as active mobile app users (57.1%; 209/366), and nearly half reported being interested in using novel mobile apps on a daily basis (45.9%; 168/366). Ethnicity information is similar to that in Study 1; either there were basically no ethnic minorities in the sample or their number was extremely small (<1%).

As with Study 1, the socioeconomic background of the sample was relatively evenly spread; 24.9% (91/366) reported their family background as *working class*, 46.7% (171/366) as *middle class*, 26.8% (98/366) as *upper middle class,* and 1.6% (6/366) as *upper class*. Of the whole sample, 23.0% (84/366) had had a diagnosis of clinical depression at some point in their lives and 5.4% were currently clinically depressed. In addition, 17.5% (64/366) of the respondents reported worse or much worse health than average people of their age, whereas 35.6% (130/366) reported better or much better health than the average person of their age. Altogether, 23.0% (84/366) of respondents reported having difficulties *quite often* or *often* in making ends meet (=not having enough money). Moreover, 12.3% (45/366) of respondents described being *rather dissatisfied* or *very dissatisfied* with their lives.

Every fifth respondent (20.8%; 76/366) had previous experience with well-being apps, and 55.5% (203/366) stated that they would be interested in using apps geared to stress management.

## Results

### Findings From Study 1

In the first questionnaire round, we investigated correlations between the Big Five personality dimensions and other stress-related variables. The correlations are presented in Multimedia Appendix 1.

Neuroticism was strongly associated with rumination, anxiety, and depressive symptoms. People with higher neuroticism reported more stress and were more likely to have a diagnosis of clinical depression. Furthermore, higher neuroticism was associated with lower happiness and being less content in the current life situation. Higher neuroticism was also associated with a history of absences from work or similar duties for psychological reasons and worry about the financial situation and was negatively associated with social status in society and personal health situation.

Extraversion had a strong negative association with anxiety, depressive symptoms, and rumination. Furthermore, extraversion seemed to be associated with a higher level of happiness in the current life situation and social status together with the prevailing health situation.

People characterized by the personality trait of agreeableness were discovered to have a lower tendency for rumination, self-reported stress, depressive symptoms, and anxiety. This personality trait appeared to also be associated with general happiness in life as well as with satisfaction with current social status, health, and financial situation.

Conscientiousness had a positive association with happiness in life, social status, and health situation. However, it was negatively associated with the perceived financial situation. This personality trait was negatively associated with rumination, anxiety, depressive symptoms, and self-reported stress.

The personality trait of openness to experience was positively associated with satisfaction with one’s life and financial situation, and interestingly, people characterized by openness appeared to be more prone to self-reported stress and absences from work owing to psychological reasons.

### Findings From Study 2

We investigated how a person’s characteristics correspond to actual interest to use stress management apps and whether it would be possible to distinguish a potential user population with specific personality traits and stress proneness. This was initially evaluated by correlating questionnaire variables with the survey question “Are you interested in using applications that help in stress management?;” significant correlations are presented in Table 2.

The correlation analysis revealed that previous stressful life events, current self-reported stress, diagnosed depression, history of absences from work for psychological reasons, and previous experience of psychological treatment were significantly related to interest for stress management app use. Thus, individuals who are stressed and depressed appear to be the most in need of the app. Furthermore, current health situation, financial situation, and perceived general happiness in the current life situation were correlated with stress management app usage interest. Stress proneness and perceived past stressful life events and current self-reported stress therefore appear to be associated with higher levels of acceptance of a stress management solution and with the interest to use the technology.

Rumination, anxiety, and depressive symptoms were significantly associated with usage interest. Individuals characterized by the Big Five personality traits of neuroticism and agreeableness appeared to have a significant positive association with interest to use stress management apps, with r=.27 and .11, respectively. The first questionnaire round already identified the strong correlation between neuroticism and the tendency for rumination and depressive symptoms, among others.

We also examined whether respondents’ general interest in using novel mobile apps on a daily basis and previous experience of using well-being apps correlated with personality and stress-related background variables. Interestingly, no significant correlations emerged between these variables.

Owing to associations with neuroticism and other personality traits, the associations between the coping styles, rumination, and other variables shown in Table 2, and the dependent variables, were not statistically significant in the combined model of logistic regression analysis (*P*>.05). The conducted analysis did not include coping styles and rumination for said reasons. The binary logistic regression analysis included standardized values for covariates of personality traits (N, E, A, C, and O), sex and age. The regression analysis showed statistically significant associations between the interest to use stress management apps and neuroticism (*P*<.001) and agreeableness (*P*=.010). When a person’s neuroticism is one SD above the mean, that is, larger than among 68% of the people, he or she has 2.036 times higher odds (95% CI 1.543 to 2.687) to be interested in using stress management apps. Respectively, when a person’s agreeableness is higher than the mean by one SD, the person has 1.379 higher odds to be interested in using stress management apps (95% CI 1.081 to 1.758). Sex did not have a significant association with the interest in using stress management apps, when analysed together with the said personality traits and age.

**Table 2:** Intercorrelations between Study 2 variables and interest to use stress management apps. Significant associations are indicated in italics.

Variable	Interest in using novel mobile apps on a daily basis		Interest in using stress management app	
	r value	*P* value	r value	*P* value
Age	−.05	.35	−.02	.78
Happiness	− *.11*	*.03*	− *.11*	*.04*
Social status	−.01	.86	−.05	.38
Financial situation	*.11*	*.03*	*.15*	*.003*
Health situation	−.06	.29	− *.16*	*.002*
Rumination	.03	.63	*.19*	*<.001*
Self-reported stress	.02	.73	*.24*	*<.001*
Stressful and hard life-event within past 5 years	.06	.22	*.16*	*.002*
Reaction to this life-event with a long-term reduction of performance	−.04	.43	− *.13*	*.01*
Absence from work for psychological reasons	.04	.44	*.17*	*<.001*
Professional help for psychological problems	.03	.56	*.18*	*<.001*
Depressive symptoms	.07	.16	*.19*	*<.001*
Anxiety	.08	.14	*.18*	*<.001*
Neuroticism	.05	.38	*.27*	*<.001*
Extraversion	.08	.15	.01	.90
Agreeableness	.00	>.99	*.11*	*.03*
Conscientiousness	− *.11*	*.04*	−.09	.09
Openness to experience	.08	.15	.01	.80

## Discussion

### Research Question 1: Personality’s Influence on Stress Proneness and Coping (Study 1)

The findings of the first questionnaire revealed that, as expected, neuroticism was strongly linked to rumination, anxiety, and depressive symptoms. Furthermore, people characterized by neuroticism experienced more self-reported stress and reported being less happy and content in their current life situation, consistent with previous research [5]. Thus, the study population was similar to other populations studied with the said variables.

Extraversion may act as a protective factor for stress-related disorders because it was associated negatively with anxiety, depressive symptoms, and rumination. This finding is also congruent with the results of Takano and Tanno [16] and Bunevicius et al [22], who showed that higher levels of extraversion are negatively associated with stress. Agreeableness was found to diminish the tendency for rumination, self-reported stress, depressive symptoms, and anxiety. Conscientiousness was associated positively with happiness in life, social status, and health situation. In addition, conscientiousness associated negatively with rumination, anxiety, depressive symptoms, and self-reported stress. Openness to experience was positively associated with satisfaction with one’s financial situation and, perhaps surprisingly, with self-reported stress and absence from work for psychological reasons. Overall, higher levels of extraversion, agreeableness, and conscientiousness were associated with lower self-reported stress, thus providing possible benefits for stress outcomes.

### Research Question 2: Stress Management App Usage Interests (Study 2)

After the initial correlation analysis, we conducted a binary logistic regression analysis with standardized variables. The results of the analysis suggested that individuals characterized by the Big Five personality traits of neuroticism and agreeableness are more likely interested to use stress management apps. People with higher neuroticism (or lower emotional stability), being more stress-sensitive, seem to be adaptive in this regard. When it comes to interest in stress management apps, their personal topics of interest coincide with their own benefit. On the contrary, higher agreeableness could increase the odds of being interested in using stress intervention software. Odds ratio between neuroticism and usage interest indicated that increase by one SD of neuroticism is associated with 2.036 times higher odds to be interested in using stress management apps. Respectively, the odds ratio between usage interest and agreeableness was 1.379. This is a particularly interesting finding as neuroticism and agreeableness were found to be negatively associated (r=−.24, *P*<.001). Thus, there are likely to be subgroups of people who would benefit from a stress management app but who are inhibited by being interested in using them by some other trait, such as a very low agreeableness.

The findings support earlier research showing that personality traits are important contributors to technology acceptance [44]. For instance, the tolerant and cooperative nature of agreeable people makes them easier to accept new technologies, in general [45]. However, the possible impact of personality traits on the adoption of mobile apps has been shown to differ between different app categories [46]. Our findings suggest that especially individuals with high agreeableness and high neuroticism are more likely to be interested in using mobile stress management solutions.

Earlier research has emphasized the importance of gaining deep insights into a person’s personality, stress proneness, and coping strategies [14]. Another consideration should be to avoid burdening the users with data collection activities, instead using the data already collected [47]. Previous studies have shown that people may avoid attending face-to-face therapy as mental health problems and illnesses are still a taboo for many people. In the future, mobile stress self-management tools could be used in combination with traditional care as people who suffer from depressive symptoms or related conditions could benefit from external motivation to encourage themselves to use self-management solutions for relieving their mental symptoms [48]. In addition, people’s attitudes toward mobile phones as monitoring and self-management tools for mental health have been shown to be positive [49,50].

The findings suggest that tailoring the content of interventions according to personality and personal needs is essential to make digital interventions more personally relevant. Greater practitioner and end-user involvement in the co-design process would help avoid a mismatch between technology and the designated context of use [51,52]. Tailoring should not be done on a superficial level but should take into account the user’s personal situation and psychological needs. Tailored messages have proven to improve intervention acceptance and efficacy, thus increasing the willingness to change and boosting treatment adherence and engagement rates [34,53].

### Limitations and Future Work

There are some noteworthy limitations in this study. As an exploratory study, the findings cannot be generalized too widely. Our respondents do not reflect the average population because the majority of the questionnaire respondents were full-time students and women aged under 30 years. To further complicate our study on the feasibility and validity of profiling survey respondents, personality appears to be more strongly associated with coping in young samples [5]. Stress coping strategies seem to change over time as responses to stress are driven more strongly by temperament in younger individuals [5]. Gender may also moderate relations between personality and coping styles owing to sex differences in the types of stressors experienced [54]. Another important aspect to consider is that the study was conducted among Finnish people. As attitudes toward stress and personality variables may differ between cultures, the findings may not be directly transferrable to other cultural settings. It is possible that the characteristics of our respondent population influenced the results, as they are by no means representative of all adults. Our results should be further investigated and confirmed in more diverse populations. In addition, multiple correlations were analyzed and presented simultaneously in the results, which might itself be somewhat problematic. On the contrary, the significances of correlations were relatively high and the results were logical and in line with each other and with previous findings, with no large outliers. Thus, the results may be considered relatively reliable in that sense.

Our future work will focus on developing a stress management app that supports users in their everyday life with personally meaningful interventions. It would be beneficial to highlight and create more personalized solutions for the groups that could benefit from stress-reduction interventions but who have other traits that make their interest less likely, such as people at the higher end of neuroticism and very low agreeableness. According to previous research, individuals characterized by different personality dimensions are accustomed to using different coping strategies. We hypothesized that after having identified the user’s personality, we could define distinct user profiles and identify preferred coping strategies and intervention(s). Thus, we plan to merge user profiles and characteristic coping styles to validate the approach of automatically determining and tailoring the content of mobile interventions.

### Conclusions

This study serves as a basis for a mobile service development process helping to derive design implications for a stress management solution that would be effective and acceptable. We studied how personality is associated with stress among Finnish university students and how personality may influence on an individual’s interest to use a self-management app for managing stress. According to our findings, individuals characterized by the personality traits of neuroticism and agreeableness are more likely to be interested to use stress management apps. These findings suggest that stress management apps should consider tailoring content based on the user’s personality or similar constructs to enhance user adherence and engagement. However, further research on how to best accomplish this is needed.

## Appendix

Multimedia Appendix 1 Intercorrelations (r value; P value) between Study 1 variables.

## Abbreviations

ERI: effort-reward imbalance
NEO-FFI: Neuroticism, Extraversion, Openness-Five-Factor Inventory
