# Supplementary material for: Influence of Personality and Differences in Stress Processing Among Finnish Students on Interest to Use a Mobile Stress Management App: Survey Study
Source: JMIR Ment Health. 2019 May 13;6(5):e10039. doi: 10.2196/10039 (PMC6707572; doi:10.2196/10039)
Supplement: Multimedia Appendix 1 [file mental_v6i5e10039_app1.pdf]

Multimedia Appendix 1. Intercorrelations (r value; *P* value) between Study 1 variables.

Variable, r value; *P* value													
	HAP	SOC	FIN	HEA	RUM	STR	ABS	CESD	GAD	N	E	A	C
HAP	-												
SOC	.33; <.001	-											
FIN	-.33; <.001	-.29; <.001	-										
HEA	.40; <.001	.22; <.001	-.27; <.001	-									
RUM	-.32; <.001	-.19; <.001	.25; <.001	-.25; <.001	-								
STR	-.38; <.001	-.21; <.001	.32; <.001	-.31; <.001	.56; <.001	-							
ABS	-.14; .001	-.13; .002	.23; <.001	-.22; <.001	.20; <.001	.29; <.001	-						
CESD	-.50; <.001	-.30; <.001	.35; <.001	-.44; <.001	.59; <.001	.61; <.001	.28; <.001	-					
GAD	-.34; <.001	-.22; <.001	.29; <.001	-.35; <.001	.52; <.001	.65; <.001	.26; <.001	.77; <.001	-				
N	-.43; <.001	-.31; <.001	.35; <.001	-.37; <.001	.74; <.001	.68; <.001	.30; <.001	.76; <.001	.69; <.001	-			
E	.36; <.001	.32; <.001	-.14; <.001	.23; <.001	-.38; <.001	-.23; <.001	-.11; .006	-.42; <.001	-.23; <.001	-.44; <.001	-		
A	.24; <.001	.13; .002	-.09; .02	.15; <.001	-.22; <.001	-.13; .001	-.04; .32	-.25; <.001	-.20; <.001	-.24; <.001	.37; <.001	-	
C	.25; <.001	.22; <.001	-.14; .000	.20; <.001	-.16; <.001	-.11; .004	-.16; <.001	-.26; <.001	-.10; .01	-.25; <.001	.30; <.001	.21; <.001	-
O	.36; <.001	.01; .72	.13; .001	-.06; .13	-.01; .80	.13; .001	.20; <.001	.04; .26	.08; .038	-.02; .71	.14; <.001	.06: .13	.01; .880

a. HAP, happiness; SOC, social status; FIN, financial situation; HEA, health situation; RUM, rumination; STR, self-reported stress; ABS, absence from work for psychological reasons; CESD, depressive symptoms; GAD, anxiety; N, neuroticism; E, extraversion; A, agreeableness; C, conscientiousness; O, openness to experience.
